# *Phlegmariurusvanuatuensis* (Huperzioideae, Lycopodiaceae) a new species from Vanuatu, re-circumscription of *P.nummulariifolius* and new combinations in *Phlegmariurus*

**DOI:** 10.3897/phytokeys.109.29359

**Published:** 2018-10-08

**Authors:** Ashley Raymond Field

**Affiliations:** 1 Queensland Herbarium, Department of Environment and Science, Mount Coot-tha Botanic Gardens, Mt Cooth-tha Road, Toowong, Queensland 4066, Australia Queensland Herbarium Toowong Australia; 2 Australian Tropical Herbarium, James Cook University, P.O. Box 6811, Cairns, Queensland 4878, Australia James Cook University Cairns Australia

**Keywords:** *
Phlegmariurus
*, Lycopodiaceae, Vanuatu, Oceania

## Abstract

*Phlegmariurusvanuatuensis* A.R.Field is described as a new species for plants endemic to the islands of Vanuatu that were previously identified with *P.nummulariifolius* (Blume) Ching. The Vanuatuan species differs from the widespread Asian-Oceanian species in several characteristics, most notably its acutely divergent leaf arrangement and thicker less branched fertile spikes. *Phlegmariurusnummulariifolius* is here re-circumscribed as plants occurring in Asia and into Oceania as far east as the Solomon Islands, being replaced eastwards by *P.vanuatuensis*. In addition, new nomenclatural combinations are made for *Phlegmariurusaustralis*, a species from Polynesia and for *Phlegmariuruscopelandianus*, a species from Malesia.

## Introduction

*Phlegmariurus* Holub is a genus including epiphytic, epilithic and terrestrial plants that inhabit perhumid forests and montane regions throughout the tropics and subtropics globally. The global diversity of *Phlegmariurus* is grouped into a predominantly Neotropical clade which has dispersed several times into Africa, Madagascar and Hawaii and a predominantly Palaeotropical clade with a single dispersal into the Neotropics (Field et al. 2016; Bauret et al. 2018; Testo et al. 2018a, b). In Asia and Oceania, almost half the species diversity belong in as-yet unresolved clades related to *P.phlegmaria* (L.) Holub, *P.phlegmarioides* (Gaudich.) A.R.Field & Bostock and *P.carinatus* (Desv.) Ching or *P.squarrosus* (G.Forst.) Á.Löve & D.Löve and *P.myrtifolius* (G.Forst.) A.R.Field & Bostock (Field et al. 2016; Bauret et al. 2018; Testo et al. 2018a, b). Within Oceania, the diversity of *Phlegmariurus* has been taxonomically reviewed only in New Guinea, the Solomon Islands, New Caledonia, Fiji and Australia (Herter 1909; Herter 1916; Alderwerelt van Rosenburgh 1917; Compton 1922; Copeland 1929a, b, 1931, 1936; Brownlie 1977; Jones and Gray 1985; Chinnock 1998; Field and Bostock 2008); but Vanuatu has been relatively neglected. Only one species, *P.oceanianus* (Herter) A.R.Field & Bostock has previously been described coming from Vanuatu, even though many other Vanuatuan specimens fit poorly into preexisting taxa from elsewhere. This paper examines the systematic placement of plants hitherto regarded as *Phlegmariurusnummulariifolius* (Blume) Ching occurring in Vanuatu and this species is herein described as new.

## Methods

Herbarium materials of *Phlegmariurus* from across Asia and Oceania were examined in AAU, B, BONN-Nessel, BR, BRI, CANB, CNS, KLU, P, PR, PRC, QRS, UC and US. Living materials were examined both in the field in Australia and Asia as well as in cultivation at James Cook University, Australia. Measurements were taken both from herbarium specimens and living specimens, and where this differs it is noted. Herbarium acronyms follow Index Herbariorum [http://sweetgum.nybg.org/science/ih/] and standard forms of author and publication citations follow International Plant Name Index [https://www.ipni.org/]. Specimens annotated ‘!’ have been physically seen, and specimens annotated ‘*’ have been seen as photographs or scans.

A proposed Conservation status was assessed against the IUCN Red List Categories and Criteria (IUCN 2012). Area of Occupancy (AOO) and Extent of Occurrence (EOO) where calculated using the GeoCAT tool (http://geocat.kew.org/Bachman et al. 2011) based upon map-assigned latitudes and longitudes extrapolated from herbarium labels and a grid cell of 10 km^2^. The centre of the island from which a specimen was selected in instances where no finer detail localities were recorded on herbarium labels.

## Taxonomy

### Phlegmariurus vanuatuensis

Taxon classificationPlantaeLycopodialesLycopodiaceae

A.R.Field sp. nov.

urn:lsid:ipni.org:names:60477108-2

**Figure 1.**
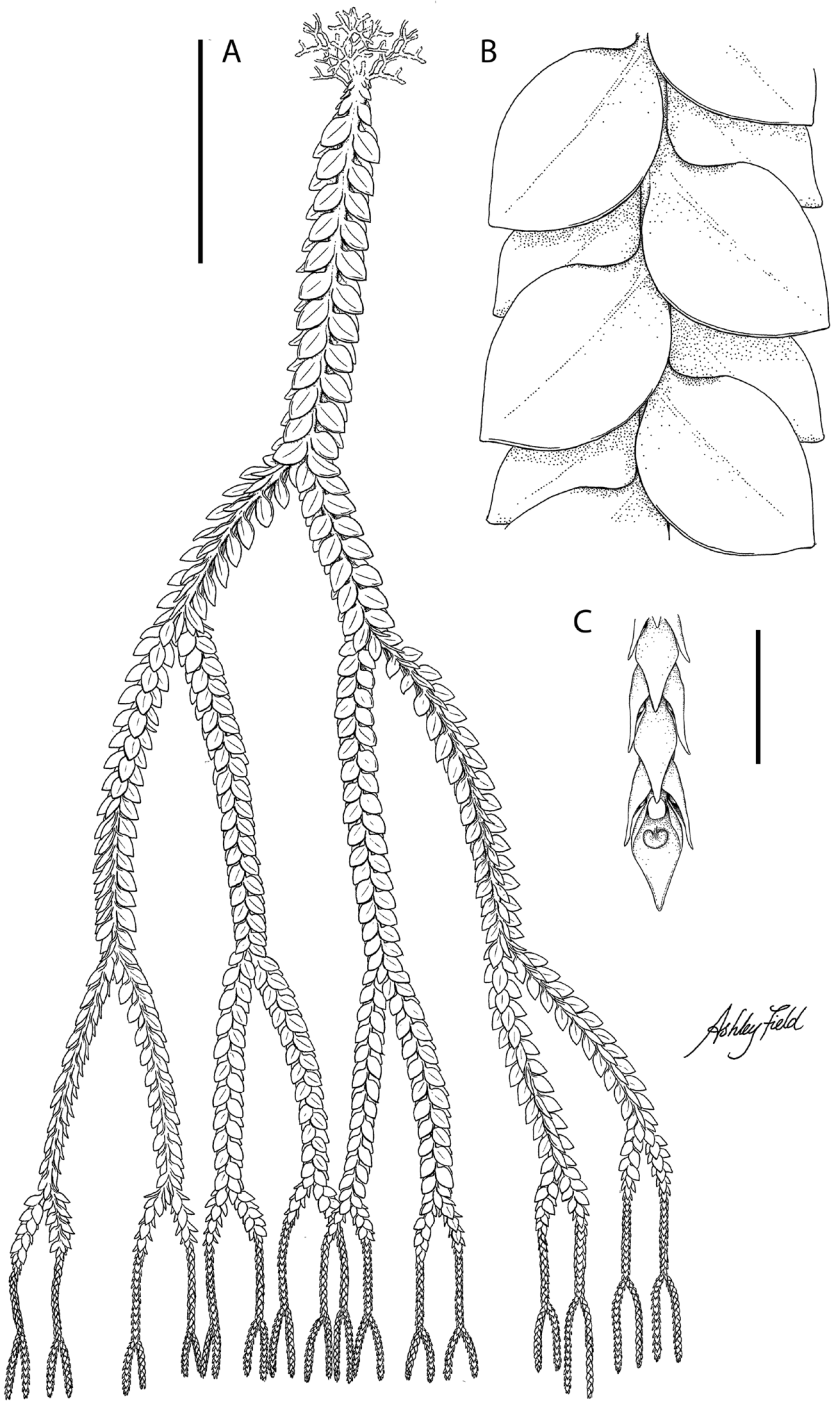
*Phlegmariurusvanuatuensis* ARF1140: **A** habit, leaf arrangement and fertile spikes **B** close up of sterile leaf arrangement showing subopposite decussate leaf arrangement and ovate-oval leaves **C** close up of fertile spike showing scale like sporophylls and a sporangium. Scale bar: 10 cm (**A**); 1 cm (**B, C**). Illustration by A.R.Field.

**Figure 2.**
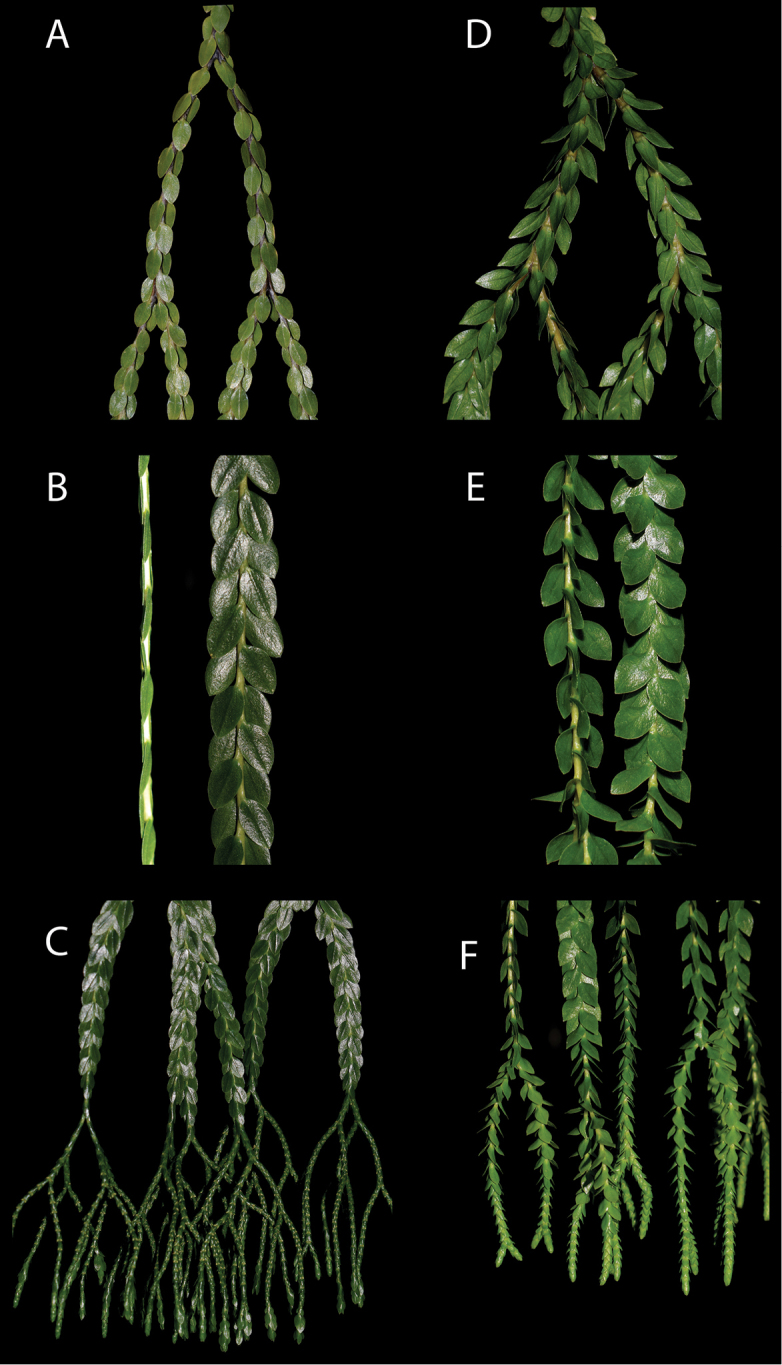
*Phlegmariurusnummulariifolius* ARF0002: **A** basal region of shoot showing branching pattern and leaf arrangement **B** medial region of shoot showing adpressed leaf arrangement in facial and lateral view **C** distal region of shoot showing abrupt transition to sporophylls and filiform fertile spikes. *Phlegmariurusvanuatuensis* ARF1140 **D** basal region of shoot showing branching pattern and leaf arrangement **E** medial region of shoot showing divergent leaf arrangement in facial and lateral view **F** distal region of shoot showing gradual transition to sporophylls and funiform-filiform fertile spikes. Photos by A.R.Field

Figure 1 A–C , Figure 2 D–F

#### Diagnosis.

*Phlegmariurusvanuatuensis* is similar to *Phlegmariurusnummulariifolius* but differs in having acutely spreading non-flattened sterile leaves (compared with adpressed and imbricate leaves flattened in on plane in *P.nummulariifolius*), thicker pale green-brown stem bases 3.5–5.5 mm diameter in *P.vanuatuensis* (compared to thinner dark black-brown stem bases 1.5–3.5 mm diameter in *P.nummulariifolius*) and a gradual transition to thicker less ramified fertile spikes 2–5.5 mm diameter in *P.vanuatuensis* (compared to an abrupt transition to slender ramified fertile spikes 1–2.5 mm in *P.nummulariifolius*).

#### Type.

Nouvelle-Hebrides: Erromango, forêt dense au N du camp du km 17, alt. 300 m, 4 Aug 1971, *J. Raynal RSNH 16213* (Holotype: P01221002!; isotype: P01219313!).

#### Description.

Sporophytes herbaceous, epiphytic, with tufted isodichotomous arching to pendulous shoots and with dichotomous roots emerging from the base of the tuft. Shoots abruptly to gradually heterophyllous; sterile sections leafy, 12–18 mm in diameter and usually 20–50 cm long, evenly branched 1–4 times; fertile sections filiform-funiform, 2–4.5 mm in diameter and up to 300 mm long, branched 0–3 times, usually unbranched at base. Stems fleshy, 2.5–5.5 mm in diameter in basal module without the leaves, pale green or light stramineous brown, and bearing indistinct longitudinal grooves between the rows of leaves. Leaves sessile, supine, decurrent, firm, orthostichous in four strict rows comprised of 2 alternating sub whorls of 2, acutely spreading, ovate to ovate-oval, 6–14.5 mm long × 6–9 mm wide, with a broad rounded base and rounded, obtusely pointed or acutely pointed apex, leaves flat to twisted with entire margins, mid glossy green to light yellow green. Leaves in the basal modules more crowded, ovate with an acute apex and with a narrowed sub-petiolate base, in median modules more widely spaced and diverging, sessile with an almost amplexicaule base, and in the distal modules transitioning in shape to sporophylls. Sporophylls gradually to sharply differentiated from sterile leaves, sessile, supine, orthostichous in two alternating pairs of two, acutely divergent to adpressed, scale like, ovate-rhomboid with a cuneate to rounded amplexicaule base and an acute apex, 1.5–5.2 mm long × 1.5–2.8 mm wide, overlapping. Sporangia borne on the upper surface in the axils of sporangia, reniform, 1–1.2 mm in diameter, mostly covered by the sporophyll. Spores isotetrahedral, 30–40 μm in diameter, with convex lateral margins, smooth proximal surfaces and moderately foveolate distal surface. Gametophytes holomycoheterotrophic, dorsiventral with paraphyses among the gametangia on the upper surface. *Vanuatu flat tassel-fern*.

#### Additional specimens examined.

VANUATU: Nouvelles Hebrides, [n.d.], *M. MacDonald 59* (P01219315). Ile de Pentecôte, zone axiale: Lalak (cote 520), 15 Sep 1936, *E. Aubert de la Rüe s.n.* (P01219311). Vate, 500–600 m, May 1965, *M. Schmid 188* (P01222833). Iles Banks, Vanua-Lava, epiphyte en forêt, 600 m, 14 Jun 1983, *P. Morat 7472* (P01238484). NW of Efate, unnamed hill N of Trig Point above Narabut camp, occasional epiphyte in forest on summit of hill, 1600 ft [488 m], 5 Jul 1971, *A.F. Braithwaite RSNH 2040* (P01219314). S. Erromanga, high epiphyte in forest, 300 ft [91 m], 6 Aug 1971, *A.F. Braithwaite RSNH 2275* (P01219309). N. Espiritu Santo, Apouna Valley, epiphytic in forest, 3000 ft [914 m], 1 Sep 1971, *A.F. Braithwaite RSNH 2355A* (P01219310). N. Espiritu Santo S.W. Bay, occasional epiphyte in ridge top forest, 1300 ft [396 m], 11 Oct 1971, *A.F. Braithwaite RSNH 2601* (P01219312). N. Espiritu Santo S.W. Bay, occasional epiphyte in ridge top forest, 1300 ft [396 m], 11 Oct 1971, *J. Raynal RSNH 16213* (P01221002). Erromango epiphyte 300 m, 4 Aug 1971, *J. Raynal RSNH 16213* (P01219313). Iles Banks, Vanua-Lava, crête vers, 500 m, 13 Jun 1983, *J.M. Veillon 5542* (P01253017). Pentecôte, Enkul, 550 m, 28 Sep 1984, *P. Cabalion 2568* (P01236864). Mallicolo, SW Bay, entre Lenbongbong et Lendemboi, 25 Sep 1986, *G. Vourdy 823* (P01238278). Efate, *M.A. Clements 5640* (CBG8916282). Cultivated James Cook University ex Efate, 30 Aug 2006, *A.R. Field 1140* (BRI, CNS).

#### Distribution, habitat and ecology.

Endemic to Vanuatu where it occurs as an uncommon epiphyte on the bark of tree trunks and branches in the canopy to subcanopy of mature trees in lowland to montane primary tropical rainforest.

#### Conservation status.

Vulnerable. *Phlegmariurusvanuatuensis* is considered eligible for IUCN listing as Vulnerable (IUCN 2012) on the basis of its Area of Occupancy being less than 20,000 km^2^. Its EOO was calculated at EOO 34,668 km^2^ and the AOO at 800 km^2^ using the GeoCAT tool. In addition, it meets criteria A.1.c. as it has experienced a catastrophic reduction in numbers and in particular loss of its intact rainforest canopy habitat following Tropical Cyclone Pam in 2015. It is also considered eligible for listing as Vulnerable on the basis of criteria B.1.a. as it has severely fragmented population scattered over disjunct islands, and is estimated to occur at fewer than 10 locations. In addition, it meets criterion B.1.b. (v & iv) as it is inferred to have experienced, and continue to experience an ongoing decline in the extent and quality of habitat and number of locations or subpopulations and number of mature individuals owing to land clearing of its habitat, and in particular owing to destruction of its habitat by severe tropical cyclones. In addition, the uniqueness of this species may make it a target for rare plant collectors.

#### Etymology.

Named for the origin of this species in Vanuatu. Vanuatu is a composition of the Austronesian words ‘*Vanua*’ meaning home or land and ‘*Tu*’ meaning stand.

#### Note.

*Phlegmariurusvanuatuensis* is closely related to *P.nummulariifolius* and has been hitherto included in that species. It appears to be the easternmost allopatric species of the *P.nummulariifolius* group, differing in several stable morphological characters. Living plants of both species are more distinct than pressed material (Figure 2), as the characteristic divergent layered leaf architecture of *P.vanuatuensis* is flattened during pressing, the thick fleshy stems collapse and colour is lost during drying (Figure 2). The lower stems of *P.vanuatuensis* are thicker, fleshier and light green or pale stramineous brown compared to *P.nummulariifolius* which has stems that are more slender, dark purplish brown and more lignified. The sterile leaves of *P.vanuatuensis* are acutely spreading, twisting slightly and being almost amplexicaule at their base compared with the tightly adpressed and uniformly planar flat leaves of *P.nummulariifolius*. The leaf colour of *P.vanuatuensis* is usually a bright green whereas in *P.nummulariifolius* it is usually a dark green. The fertile spikes of *P.vanuatuensis* are funiform and relatively unbranched compared with being filiform and multibranched in *P.nummulariifolius*. The transition from sterile to fertile spikes is more gradual than for *P.nummulariifolius*. Young plants of both species are very similar but can usually be differentiated on the basis of leaf divergence.

The diagnostic traits of *Phlegmariurusvanuatuensis* appear to be consistent across its population whereas the traits of *Phlegmariurusnummulariifolius* appear to be consistent throughout its Malesian, New Guinean and Solomon Island range with plants as close as the Santa Cruz Islands typical of *P.nummulariifolius*. It is expected that *P.vanuatuensis* has dispersed eastward from *P.nummulariifolius*, or a shared ancestor, but has become isolated in the islands Vanuatu leading to allopatric divergence there. Alternatively, it could have colonized Vanuatu and hybridized with *P.phlegmarioides*, as it also shares some traits with that species such as the divergent leaf planes, layered arching branches and the thicker stems and fertile spikes.

*Phlegmariurusvanuatuensis* can be readily differentiated from *P.delbrueckii* (Herter) A.R.Field & Bostock by its larger size, and by having terete fertile spikes rather than the quadrangular fertile spikes found in *P.delbrueckii*. It can be readily distinguished from *P.phlegmarioides* by its leaves being supine and somewhat more flattened in one plane, rather than radiating in four planar ranks as found in *P.phlegmarioides*. Another species occurring further east in Polynesia, *P.ribourtii* (Herter) A.R.Field & Bostock has thicker and distinctly quadrangular fertile spikes and more lingulate-oval leaves with a different arrangement.

*Phlegmariurusvanuatuensis* is remarkably convergent with the unrelated broad leafed form of *P.obtusifolius* (P.Beav.) A.R.Field & Bostock *s.l.* [=*P.pachyphyllus* (Kuhn ex Herter) A.R.Field & Testo *s.s.*] which occurs in a similar habitat in Madagascar and islands of the West Indian Ocean. Together, these species repeat a general trend in which several unrelated species of *Phlegmariurus* occurring epiphytically in lower altitudes of offshore oceanic islands have broader leaves with rounded apices.

### Phlegmariurus nummulariifolius

Taxon classificationPlantaeLycopodialesLycopodiaceae

(Blume) Ching, Acta Bot. Yunnan. 4(2): 125 (1982)

≡Lycopodiumnummulariifolium Blume, Enum. Pl. Javae 2: 263 (1828). Urostachysnummulariifolius (Blume) Herter, Philipp. Sci. 22: 182 (1923). Huperzianummulariifolia (Blume) Chambers, Jermy & Crabbe, Brit. Fern Gaz. 10: 176 (1971). **Type citation.** ‘Crescit Javae montosis ex arboribus pendulum’. **Type.** Sallak [Java], *C.L.Blume s.n.* (lectotype, designated here: L0057363* [L 908.342-266]; isolectotypes/syntypes: L 908.326-17 *n.v.*, NY00127360*). **Type note.**Øllgaard (1989 p. 59) lists two syntypes in L, and a third duplicate of a Blume gathering is known in NY. The most complete specimen in L is selected as the lectotype, the other having not been relocated. **Etymology.** Named in reference to the round coin like leaves.
=Lycopodiumrotundifolium Roxb., Calcutta J. Nat. Hist. 4: 473 (1844). **Type citation**. ‘*Wallich* cat. 65, no. 2183’ and ‘*Nat.* of *Sumatra*, and a most beautiful species it is.’. **Type.** [s.l., n.d., s.c. s.n. (plant labelled in Roxburgh’s hand] (? holotype [see Morton 1973 p. 328–329]: E00429111* [E-Greville]). **Etymology.** Named in reference to the round leaves.

#### Description.

Sporophytes herbaceous, epiphytic, with tufted isodichotomous pendulous shoots and with dichotomous roots emerging from the base of the tuft. Shoots abruptly heterophyllous; sterile sections leafy, 5–12 mm in diameter and usually 20–250 cm long, branched 1–6 times being relatively unbranched basally and more frequently ramified distally; fertile sections filiform, 1–2.2 mm in diameter and up to 400 mm long, multibranched 1–6 times. Stems slender and woody, 1.5–3.5 mm in diameter in basal module without the leaves, light green to dark purplish-brown, and bearing indistinct longitudinal grooves between the rows of leaves. Leaves sessile, supine, decurrent, firm, orthostichous in four strict rows comprised of 2 alternating sub whorls of 2, adpressed and imbricate, ovate-oval, 6–14 mm long × 4–7.5 mm wide, with a rounded base and similar rounded apex, leaves flat with entire margins, dark glossy green to light yellow green. Basal leaves sometimes more scale like and leaves in basal modules sometimes lanceolate-ovate and more widely spaced out on elongated naked stems, leaves in median and distal regions ovate-oval. Sporophylls markedly differentiated from sterile leaves, sessile, supine, orthostichous in two alternating pairs of two, scale like, adpressed throughout, ovate-rhomboid with a cuneate base and acute apex, 2.1–3.5 mm long × 1.4–2.2 mm wide, overlapping or occasionally spaced apart with stem visible between subwhorls of sporophylls. Sporangia borne on the upper surface in the axils of sporangia, reniform, 0.8–1 mm in diameter, mostly covered by the sporophyll. Spores isotetrahedral, 30–40 μm in diameter, with convex lateral margins, smooth proximal surfaces and moderately foveolate distal surface. Gametophytes holomycoheterotrophic, dorsiventral with paraphyses among the gametangia on the upper surface. *Flat tassel-fern* [Australia], *Kied Hoy* [Thailand], *Pum-borey* [Bougainville Island], *Yúlín* [Singapore]. Figure 2A–C.

#### Specimens examined.

ASIA. VIETNAM. Tonkin: Saigon, [n.d.], *Faurie 182* (BONN-Nessel 646b). INDONESIA. Java: [s.l.], 1869, [s.c.] *13* (P1238007). Enviriv du Jardin Botanique du Buitenzorg, Jan-Feb 1877, *H.de Poli [s.n.*] (P1596127). Java, 1854, *M.Göring 210* (P1238008; P1238006). Java, [n.d.], *Kollmann [s.n.*] (P1238019). Gedeh Gebirge, 1910, *Königsberger [s.n.*] (BONN-Nessel 642b). [s.l.], [n.d.], *Schneider [s.n.*] (P1220849). Java, [n.d.], *C.Treub [s.n.*] (P1238013). [s.l.], [n.d.], *Zollinger [s.n.*] (BONN-Nessel 642a). Java, [n.d.], *Zollinger [s.n.*] (P1238004). [s.l.], [n.d.], *Zollinger [s.n.*] (P1238014). Java, [n.d.], *Zollinger 175-Z* (P01238005; P01238012; P01238025). Kalimantan: W. KokLai [illeg.], 800 m, 12 Sep 1925, *F.H. Enderl 3804* (BONN-Nessel 646a). Gunung Samarinda, Ost-Borneo, Aug 1910, *E.Rŭdel [s.n.*] (BONN-Nessel 643). Sumatra: Island of Siberut, 8 Sep 1924, *C. Boden-Kloss 10581* (US1346685). Siberut, 8 Sep 1924, *C. Boden-Kloss 10581* (US1346686). Enggano, Boea-boea, 100 m, 29 May 1936, *W.J. Lutjeharms 3874* (US2139904). East Coast, Vicinity of Aek Moente, 500 m, 15 Jun-9 Jul 1936, *Rahmat Si Boeea 9168* (US2717673). Province indet: Archipel. Ind. ?, [n.d.], [*s.c.] 14* (P1220851). Archipel. Ind., [n.d.], [*s.c.] 219* (P1220850). Ostindien, [n.d.], *Schneider [s.n.*] (P1220852). Boesoe Nal’ Besie, May 1921, *Toxopeus 181* (P1220854). MALAYSIA. Johore: Gunung Janing Plateau, 430 m, 23 Oct 1985, *K.M. Wong FRI 30916* (QRS113912). Kedah: B. Blukang Parang, G. Bintang, 16 Apr 1928, *Haniff Singapore Field # 21065* (UC346255; US1704974). Kelantan: Gua Panjang, Gua Nimik, 305 m, 21 Oct 1927, *M.R. Henderson 19534* (US1274282). Suga Keteh, 16 Feb 1924, *Nur 12108* (UC234463). Negri Sembilan: Ulu Langat, Stream side vegetation along Sungei Kenaboi, about 2 miles downstream of Sungei Damar junction. Kenaboi Forest Reserve, 610 m, 20 May 1976, D.W. Lee UL-61* (P1266534; US2920343). Pahang: S.Sat., Pahang, 29 Aug 1929, *M.R. Henderson 22091* (UC462868). Malay Peninsula, S. Sat, 19 Jul 1929, *M.R. Henderson Singapore Field # 20091* (US1526658). Malay Peninsula. Kuala Teku, 152 m, 21 Jul 1936, *Kiah Singapore Field # 31750* (US1703674). Malaya, Near Tunnel Rd., Penang Hill, 610 m, 4 Mar 1956, *B.E.G. Molesworth-Allen 2765* (US2255844). Penang, [n.d.], *H. Norris [s.n.*] (P1220855). Perak: Perak, [n.d.], *Scortechini [s.n.*] (BONN-Nessel 644). Sabah: Mt Kalawat, Oct-Dec 1915, *M.S. Clemens 11144* (UC211777). cultivated ex Sabah, Malaysia, 25 Apr 2018, *A.R. Field 4670* (CNS). G. Lumaku, 300 m, 7 Mar 1969, *H.P. Nooteboom 1212* (US2951438). Sarawak: [s.l.], Jul 1865, *O. Beccari [s.n.*] (US1918110). Gilam Bakun, 23 Aug 1954, *W.M. Brooke 9077* (US2292818). Bakelalan, 914 m, 23 Aug 1955, *Collector illegible 10486* (US2290680). Foot of Mt Santubong, 16 Mar 1914, *collectuer indigene 97* (P1238015; P1238016). cultivated ex Sarawak, Malaysia, 25 Sep 2016, *A.R. Field 2785* (CNS). Borneo, 17 Jun 1894, *E. Langlassé 57* (P1238009; P1238010; P1238017). Borneo, [n.d.], *Native collector 58* (US1173888). Sarawak, [n.d.], *Native collector 774* (P1238018; US1174046). Sarawak, [n.d.], *Native collector 1547* (UC218747; US1174144). Sarawak, Feb-Jun 1914, *Native collector 2143* (UC218752). Terengannu: Malay Penins.: Bukit Besar, 914 m, May 1899, *D.T. Gwynne-Vaughan 425* (US1506242). Ind. Or., [n.d.], [*s.c.] [s.n.*] (P1220856). OCEANIA. INDONESIA. West Papua: 6 km SW of Bernhard Camp Idenburg River, 1200 m, Feb 1939, *L.J. Brass 12837* (BRI327894). Mt Nettoti, Tamrau Range, 1460 m, 28 Oct 1954, *P. van Royen 3899* (P1238493; UC40507). Bernhard Camp Idenburg River, 50 m, Apr 1939, *L.J. Brass 14079* (BRI327897). PAPUA NEW GUINEA. Central Province: cultivated ex Brown River, Papua New Guinea, 3 Feb 2001, *A.R. Field 2* (CNS). Madang Province: Bundi Mission, 1300 m, 19 Jul 1992, *P.I. Forster PIF10979* (BRI549514). Manus Province: Admiralty Islands, Tingau #1, Number One Road, Manus Island, 10–12 Jul 1946, D.F. Grether 4522 (UC729030; US1918392). Morobe Province: New Guinea, Umi River, Markham Valley, 480 m, 17 Nov 1959, *L.J. Brass 32571* (US2358020). Nordöstliches New-Guinea, Morobe-Distrikt, Sattleberg, 3300 ft, 1 Feb 1936, [*M.S.] Clemens 1840* (P1242270). Charles Luis Gebirge, 1911, *Harking [s.n.*] (BONN-Nessel 655b). Waria River below Garaina, 3300 ft, 20 Jun 1962, *T.G. Hartley TGH10376* (BRI149761). Sattleberg, 600 m, Jan 1899, *E. Nyman [s.n.*] (BONN-Nessel 655a). Kaiser-Wilhelmsland, 2 Aug 1907, *R. Schlechter 16352* (P1238020; P1238022; US2990287). New Guinea. Tiaure Village, 5 miles S. of Garaina, Lae sub-district, 488 m, 22 Jul 1970, *H. Streimann NGF 45048* (US2613636). Nawata Banda, 720 m, 30 Jun 1966, *H. Streimann NGF27818* (BRI149765). Between Kaisenik and Wuri-Wuri, 1140 m, 18 Oct 1968, *H. Streimann NGF39129* (BRI149995). Tiaure Village 5 M S of Garaina, 480 m, 22 Jul 1970, *H. Streimann NGF45048* (BRI365353). Lae, FRI Botanical Gardens, 10 m, 5 Jul 1993, *W. Takeuchi 9005* (US3633126). Kikiepa Village near Wantoat, 1500 m, 5 Jun 1960, *J.S. Womersley NGF12728* (BRI149760). New Ireland Province: Namatanai sub-province; Hans Meyer Range, 625 m, 9 Oct 1975, *M.J. Sands 2169* (US3685033). North Solomons Province: Pavairi, 810 m, 20 Jan 1967, *C.E. Ridsdale NGF31021* (BRI149971). North Solomons Province: Kupei Gold Field., 1000 m, 24 Apr 1930, *S.F. Kajewski 1777* (P1219318). Bougainville Island, Kugumaru, Buin, 150 m, 18 Jul 1930, *S.F. Kajewski 1952* (P1219317; UC542111). Territory of New Guinea, Near Korpei village, 11 miles southwest of Keita, 670 m, 2 Nov 1961, *D.H. Nicolson 1533* (US2416100). Near Korpei village, 11 miles southwest of Keita, 670 m, 2 Nov 1961, *D.H. Nicolson 1544* (P1243720). Vicinity of Aku village, c. 9 miles west of Buin Station, 30 m, 23 Sep 1964, *R. Schodde 4116* (US2577448 A). Oro Province: Kokoda, 360 m, 29 Jul 1964, *A.N. Millar NGF 23563* (BRI149759). Province indet: Loganeng, [n.d.], *M.G. Balmer 54* (P1238011; P1238023; UC391741). Nouvelle-Guinee allemande, Loganeng, auf Baumen, [n.d.], *M.G. Bamler 54* (US3030998; US3031008). Bella Vista, Dieni [illeg.], 1933, *L.J. Brass 3792* (BONN-Nessel 642c). SOLOMON ISLANDS. Kolambangara: New Georgia Group, Kolombangara Island. West coast, inland from Iri iri Village (Merusu Cove), 610 m, 28 Sep 1963, T.C. Whitmore BSIP 2148 (US2577636 A). South Kolambangara, 4000 ft, 2 August 1965, *A.F. Braithwaite 411* (P1219316). Sancristobal: E. Sancristobal. Confluence of Warahite and Pagato Rivers, 150 ft, 25 Jul 1965, *A.F. Braithwaite 4175* (P1219319). Santa Cruz Islands: Vanikoro Island, 100 m, 13 Nov 1928, *S.F. Kajewski 621* (UC422664; US1758589). Ngarabu camp to end road then follow ridge, Vanikoro island, Santa Cruz province, 120–600 m, 17 Jun 2016, *C.-W. Chen SITW10583* (CNS). Santa Ysabel: Dedeu R. S.W. Santa Ysabel, 43 m, 21 Apr 1966, *Beer’s collectors BSIP 6686* (US2691129). Santa Ysabel: nr. Maringe Lagoon, Mt. Sasari, 853 m, 26 Oct 1963, *T.C. Whitmore BSIP 2429* (US2577645 A). Guadalcanal: Mt. Gallego, Guadalcanal Island, 400–1000 m, 30 Jun 2014, *C.-W. Chen SITW5240* (CNS). Hut to Mt. Tepalamenggutu, 600–1500 m, 15 Oct 2012, *T.-Y. Yang SITW1018* (CNS).

#### Distribution, habitat and ecology.

Widespread in Malesia and western Oceania where it occurs in New Guinea and the Solomon Islands as far east as the Santa Cruz Islands. A canopy and subcanopy epiphyte in lowland to montane tropical rainforest. This species often grows from decaying nests of other epiphytes, rotting logs and tree hollows but also will grow on bark on tree branches, especially where humus has accumulated.

#### Notes.

*Phlegmariurusnummulariifolius* is here circumscribed as plants occurring throughout Malesia, New Guinea and the Solomon Islands that have uniformly flattened shoots comprised of decurrent imbricate leaves, excluding plants previously identified with *P.nummulariifolius* that occur in Vanuatu which have divergent leaves and are described below as a new species. *Phlegmariurusnummulariifolius* is a widespread and remarkably uniform species throughout its range that is readily identified by its unique leaf shape and arrangement and flattened shoots on very long slender stems.

Examination of G.H.Brownlie determinations on herbarium specimens indicates that reports of *P.nummulariifolius* in Fiji (Brownlie, 1977) are based on misidentifications of *P.delbrueckii* (Herter) A.R.Field & Bostock [e.g. *Brownlie 1835* (AAU! & CHR341130] and juvenile specimens of the Fijian population of *P.phlegmarioides* (Gaudich.) A.R.Field & Bostock *s.l.* [=*P.pseudophlegmaria* (Kuhn) A.R.Field & Testo *s.s.*], both of which also have rounded leaf apices. I have not encountered any material of *P.nummulariifoliuss.s.* south or east of the Sant Cruz Islands, Solomon Islands.

The determination ‘*Lycopodium kempterianum Schlecht. n. sp.* Schlechter’ appears on the gathering ‘Neuguinea: Kaiser-Wihlems-Land, 2 Aug 1907, *R. Schlechter 16352* (BONN-Nessel 646a!, P01238020!, US2990287!), and a citation in Nessel (1939 p. 254). Schlechter’s specimen is typical of *P.nummulariifolius* and it is presumed that he did not proceed with publication of a new taxon.

##### New combinations in *Phlegmariurus*

Recent combinations and descriptions of *Phlegmariurus* bring the number of combinations in the genus globally to 288 (Hsieh et al. 2012; Øllgaard 2012a, b; Field and Bostock 2013; Øllgaard 2015; Øllgaard 2016; Arana 2016; Field et al. 2016; Duy et al. 2016; Testo 2017; Kiew and Kamin 2018; Bauret et al. 2018; Øllgaard 2016). Although the *Phlegmariurus* species diversity of the Neotropics have been comprehensively reviewed by Øllgaard (1992, 2012a, b, 2015, 2016), the species occurring in the Palaeotropics have not been as intensively reviewed. Many Palaeotropical taxa are poorly defined, being either mixed assemblages of convergent species (e.g. species in the polyphyletic *P.phlegmaria* group, Field et al. 2016) or multiple taxa described for extremes of an apparently continuously variable species (e.g. species in the *P.macgregorii* group). The two following combinations are for more clearly definable species.

### Phlegmariurus australis

Taxon classificationPlantaeLycopodialesLycopodiaceae

(Willd.) A.R.Field comb. nov.

urn:lsid:ipni.org:names:77190776-1

≡Lycopodiumaustrale Willd., Sp. pl., Ed. 4 [Willdenow] 5: 11 (1810). Lycopodiumphlegmariavar.australe (Willd.) Domin, Biblioth. Bot. 89(4) (1928). Urostachysaustralis (Willd.) Herter ex Nessel, Bärlappgewächse 224 (1939). Huperziaaustralis (Willd.) Holub, Folia Geobot. Phytotax. 20: 70. (1985). **Type citation.** ‘Habitat in societas insulus (v.s.)’. **Type.** societas insulis [Tuha’a Pae or Austral Islands, Society Islands, French Polynesia, Aug1773, *J.R.Foster & G.A.Foster s.n.*] (lectotype, designated by Øllgaard 1989 p. 33: B-W 19341-01!; isolectotypes/syntypes: GOET01283*, GOET012844*, LE00008976*). **Etymology.** Named for the southern origin of the type gathering.
=Lycopodiumsubtrifoliatum Brownlie, Nova Hedwigia Beih., 55 (Pterid. Fl. Fiji) 24 (1977). Huperziasubtrifoliata (Brownlie) Holub, Folia Geobot. Phytotax. 26(1): 93 (1991). Phlegmariurussubtrifoliatus (Brownlie) A.R.Field & Bostock, PhytoKeys 20: 48 (2013). **Type.** Mt. Korobaba, Viti-Levu, Fiji, 20 Jun 1971, *G.H.Brownlie 1287* (holotype: CHR20341227*). **Etymology.** Named for its smaller size compared to L.trifoliatum Copel.

#### Note.

*Phlegmariurusaustralis* (Willd.) A.R.Field is an earlier name for the Polynesian black-stem tassel-fern recognised by Field and Bostock (2013) as *P.subtrifoliatus* (Brownlie) A.R.Field & Bostock. It occurs in Polynesia, where it is the sole, and easternmost, vicariant of the *P.phlegmaria* group. It also occurs in eastern Melanesia and in sympatry with *Phlegmariurustrifoliatus* (Copel.) A.R.Field & Bostock in Fiji. *Phlegmariurusaustralis* can be distinguished by its shorter ovate leaves and smaller shoot diameter compared with the longer lanceolate leaves and larger overall size of *P.trifoliatus*.

### Phlegmariurus copelandianus

Taxon classificationPlantaeLycopodialesLycopodiaceae

(R. C.-Y. Chou & Bartlett) A.R.Field comb. nov.

urn:lsid:ipni.org:names:77190774-1

≡Lycopodiumcopelandianum R. C.-Y. Chou & Bartlett, Bull. Torrey Bot. Club 74: 369 (1947). Huperziacopelandiana (R.C.-Y. Chou & Bartlett) Holub, Folia Geobot. Phytotax. 26(1): 92 (1991). Lycopodiumpetiolatum Copel., Univ. Calif. Publ. Bot. 14: 377 (1929) [non Herter 1909]. **Type.** Berastagi Karoland, Sumatra, 1–5 Feb 1926, *H.H.Bartlett 6575* (holotype: UC349249!; isotype: MICH1287188*). **Etymology.** Named in honour of American botanist Edwin Bingham Copeland (1873–1964).

#### Note.

*Phlegmariuruscopelandianus* is a rare species that is endemic to Indonesia where it is an epiphyte in montane rainforest. It was placed in the group of the New Guinea endemic *P.macgregorii* (Copel.) A.R.Field & Bostock by Øllgaard (1989), but does not appear to have a close relative and has not yet been placed in molecular phylogenetic analyses. It is unique among *Phlegmariurus* in having minute, distinctly petiolate, almost spoon like leaves with a cupped apex and reflexed lateral margins.

## Supplementary Material

XML Treatment for Phlegmariurus vanuatuensis

XML Treatment for Phlegmariurus nummulariifolius

XML Treatment for Phlegmariurus australis

XML Treatment for Phlegmariurus copelandianus
